# Cynarin attenuates LPS-induced endothelial inflammation via upregulation of the negative regulator MKP-3

**DOI:** 10.1080/19768354.2022.2077438

**Published:** 2022-05-20

**Authors:** Da Bin Kim, Banzragchgarav Unenkhuu, Grace Jisoo Kim, Seung-Woo Kim, Hong Seok Kim

**Affiliations:** a Department of Molecular Medicine, College of Medicine, Inha University, Incheon, Republic of Korea; b Program in Biomedical Science and Engineering, College of Medicine, Inha University, Incheon, Republic of Korea; c Yongsan International School of Seoul, Seoul, Republic of Korea; d Department of Biomedical Sciences, College of Medicine, Inha University, Incheon, Republic of Korea

**Keywords:** Cynarin, endotoxin, endothelial inflammation, MKP-3

## Abstract

Clinical observations have revealed that non-resolving low-grade inflammation is linked to the pathogenesis of chronic inflammatory diseases, for example arthritis, atherosclerosis, Alzheimer’s disease, diabetes, and chronic kidney disease. Interestingly, low levels of circulating lipopolysaccharides (LPS) derived from the outer membrane of gram-negative bacteria appear to be one of the primary causes of persistent low-grade inflammation. The inner surface of the blood vessels is lined with endothelial cells; therefore, even low levels of circulating LPS can directly activate these cells and elicit specific cellular responses, such as an increase in the expression levels of cell adhesion molecules and proinflammatory mediators. In endothelial cells, LPS exposure results in an inflammatory response through activation of nuclear factor-kappa B (NF-κB) and mitogen-activated protein kinases. Cynarin, a phytochemical found in artichokes, has several pharmacological properties against endothelial inflammation. In the present study, we discovered that cynarin suppressed the LPS-induced increase in the expression levels of vascular cell adhesion molecule-1 and proinflammatory mediators such as monocyte chemoattractant protein-1 (MCP-1), tumor necrosis factor-α (TNF-α), and interleukin-1β in EA.hy926 cells. Further, cynarin inhibited the activation of p38 and NF-κB pathways by inducing the negative regulator mitogen-activated protein kinase phosphatase 3 (MKP-3) in LPS-stimulated EA.hy926 cells. In conclusion, cynarin alleviates inflammation by upregulating MKP-3, a negative regulator of p38 and NF-κB, and it may be a therapeutic option for treating endothelial inflammation-related diseases.

## Introduction

The term ‘low-grade endotoxemia’ was recently coined to describe subclinically elevated circulating blood endotoxin levels (Terawaki et al. 2010). It is closely correlated with chronic inflammatory diseases such as type 2 diabetes, chronic kidney disease, non-alcoholic fatty liver disease, and cardiovascular disease (Minihane et al. 2015). In addition to localized or persistent infections, leaky intestinal barriers are potential sources of blood endotoxins. Low-dose endotoxemia is more likely to develop in people with unhealthy lifestyle choices, such as smoking (Glaros et al. 2007), drinking (Rao 2009), high-fat diets (Mohammad and Thiemermann 2020), as well as in people with HIV infection (Palmer et al. 2016) or liver cirrhosis (Raparelli et al. 2017); ageing is also a predictor for low-grade endotoxemia (Goto et al. 1994; Jin et al. 2020).

Endothelial cells form the interior surface of blood vessels (Dauphinee and Karsan 2006). Gram-negative bacterial endotoxins such as lipopolysaccharides (LPS) cause inflammation in endothelial cells by directly stimulating the vascular endothelium and inducing several cellular responses, such as elevating the expression levels of cell adhesion molecules and proinflammatory cytokine/chemokines (Bierhaus et al. 2000). Such endotoxins also increase endothelial permeability and recruit monocytes and macrophages, enhancing inflammation (Cohen 2002).

Mitogen-activated protein kinases (MAPKs) are a family of serine/threonine kinases that react to a variety of stimuli, including mitogens, proinflammatory cytokines, and environmental stress (Kyriakis and Avruch 2001). While extracellular signal-related kinases (Erk) and big MAPK-1 are predominantly involved in growth and cytoprotection, Jun amino-terminal kinases (JNK) and p38 proteins play critical roles in the inflammatory and stress response (Hoefen and Berk 2002). In addition to MAPK activation, the nuclear factor-kappa B (NF-κB) pathway is vital for endothelial inflammation (Oates 2015). Cytoplasmic NF-κB activation is controlled by inhibitors of kappa B (IκB). Extracellular proinflammatory stimuli cause IκB to be phosphorylated and degraded; as a result, free NF-κB is translocated to the nucleus, stimulating the expression of downstream genes (Karin and Ben-Neriah 2000). Importantly, we recently discovered that endotoxin-induced activation of p38 and NF-B, both of which play important roles in endothelial inflammation, is suppressed by MKP-3 (Unenkhuu et al. 2021).

Cynarin, also known as 1,3-O-dicaffeoylquinic acid, is a phytochemical found in artichokes, which has antioxidant (Topal et al. 2016), antihypertensive (Hakkou et al. 2017), anti-atherosclerotic (Li et al. 2004), anti-HIV (McDougall et al. 1998), anti-carcinogenic (Zhou et al. 2020), and cholesterol-lowering properties (Chen et al. 2018). Despite its anti-inflammatory effects, little is known about how cynarin affects the inflammatory responses in endotoxin-stimulated endothelial cells.

Therefore, in this study, we explored the effects of cynarin on the inflammatory response in EA.hy926 human endothelial cells treated with LPS. Furthermore, we examined the role of the p38/NF-κB pathway and cynarin-regulated MKP-3 expression in endothelial cells. This study sheds light on the molecular processes underlying the anti-inflammatory properties of cynarin, which will aid the development of drugs to prevent and treat low-grade endotoxemia-induced endothelial inflammation.

## Materials and methods

### Reagents

LPS was purchased from Sigma-Aldrich (St. Louis, MO, USA). Cynarin was obtained from MedChemExpress (Monmouth Junction, NJ, USA).

### Cell culture

Human endothelial cells (EA.hy926; ATCC CRL-2922™) were cultured in DMEM (HyClone Laboratories, Logan, UT, USA) supplemented with 10% heat-inactivated fetal bovine serum (HyClone Laboratories) and 1% penicillin/streptomycin (WELGENE, Gyeongsan, Republic of Korea) at 37 °C in humidified 5% CO_2_ atmosphere.

### Cell viability assay

EA.hy926 cells were seeded at a density of 1 × 10^4^ cells/well in 96-well plates. A Cell Counting Kit-8 (CCK-8; Dojindo Molecular Technologies, Rockville, MD, USA) assay was used to assess cell viability 24 h after incubation with cynarin at various concentrations (0–10 μM), following the manufacturer’s instructions. The relative viability of cynarin-treated cells was measured at 450 nm using a Multiskan GO microplate spectrophotometer (Thermo Fisher Scientific).

### Western blotting

Western blotting was performed as previously described (Unenkhuu et al. 2021). The primary antibodies used in the experiments were as follows: vascular cell adhesion molecule-1 (VCAM-1; diluted at 1:1,000, Cell Signaling Technology, Danvers, MA, USA), phospho-NF-κB p65 (serine 536 [Ser536], diluted at 1:1,000, Cell Signaling Technology), NF-κB p65 (diluted at 1:1,000, Cell Signaling Technology), phospho-p38 MAPK (diluted at 1:1,000, Cell Signaling Technology), p38 MAPK (diluted at 1:1,000, Cell Signaling Technology), β-actin (diluted 1:2,000, Santa Cruz Biotechnology, Dallas, TX, USA), and MKP-3 (diluted 1:1,000, Abcam, Cambridge, UK). The HRP-conjugated secondary antibodies (1:5,000 dilution) obtained from Santa Cruz Biotechnology were used for 1 h at room temperature. Signals were detected via enhanced chemiluminescence using a ChemiDoc imaging system (Bio-Rad, Hercules, CA, USA).

### Rna isolation, reverse transcription, and real-time qPCR

Total RNA extraction from EA.hy926 cells was performed using an AccuPrep Universal RNA Extraction Kit (Bioneer, Daejeon, Republic of Korea) according to the manufacturer’s instructions. AccuPower CycleScript RT PreMix (Bioneer) was used to conduct reverse transcription after RNA isolation and quantitation. Gene expression at the mRNA level was determined via real-time quantitative polymerase chain reaction (RT-qPCR) using an AccuPower 2X GreenStar qPCR Master Mix (Bioneer) and a CFX Connect Real-Time PCR detection system (Bio-Rad). The expression of 18S rRNA was used as an internal control. Primer sequences used for RT-qPCR are listed in Table 1.

**Table 1.** Primer sequences for real-time quantitative PCR.

Template	Forward Primer	Reverse Primer
VCAM-1	5′-GATTCTGTGCCCACAGTAAGGC-3′	5′-TGGTCACAGAGCCACCTTCTTG-3
MCP-1	5′-AGAATCACCAGCAGCAAGTGTCC-3′	5′-TCCTGAACCCACTTCTGCTTGG-3′
TNF-α	5′-CTCTTCTGCCTGCTGCACTTTG-3′	5′-ATGGGCTACAGGCTTGTCACTC-3′
IL-1β	5′-CCACAGACCTTCCAGGAGAATG-3′	5′-GTGCAGTTCAGTGATCGTACAGG-3′
18S rRNA	5′-AACCCGTTGAACCCCATT-3′	5′-CCATCCAATCGGTAGTAGCG-3′

### Immunofluorescence staining

After treatment with cynarin and LPS, the EA.hy926 cells were fixed in 4% paraformaldehyde in phosphate-buffered saline (PBS) for 15 min, permeabilized with 0.3% Triton X-100, and blocked with 2% bovine serum albumin in PBS for 1 h. Following incubation with rabbit anti-NF-κB p65 at 4 °C overnight, the cells were incubated with Alexa Fluor 488-conjugated goat anti-rabbit IgG (Thermo Fisher Scientific) for 1 h at room temperature. Nuclei were stained with Hoechst 33258. Images were acquired using a fluorescence microscope (EVOS FL Cell Imaging System).

### Statistics

All data are presented as the mean value and standard error of the mean (SEM) of n measurements. Statistical comparisons of the results were performed using one-way analysis of variance. Differences were considered statistically significant when the *p*-value was less than 0.05.

## Results

### Cynarin decreases the expression levels of VCAM-1 and inflammatory cytokines in LPS-stimulated endothelial cells

**Figure 1.**
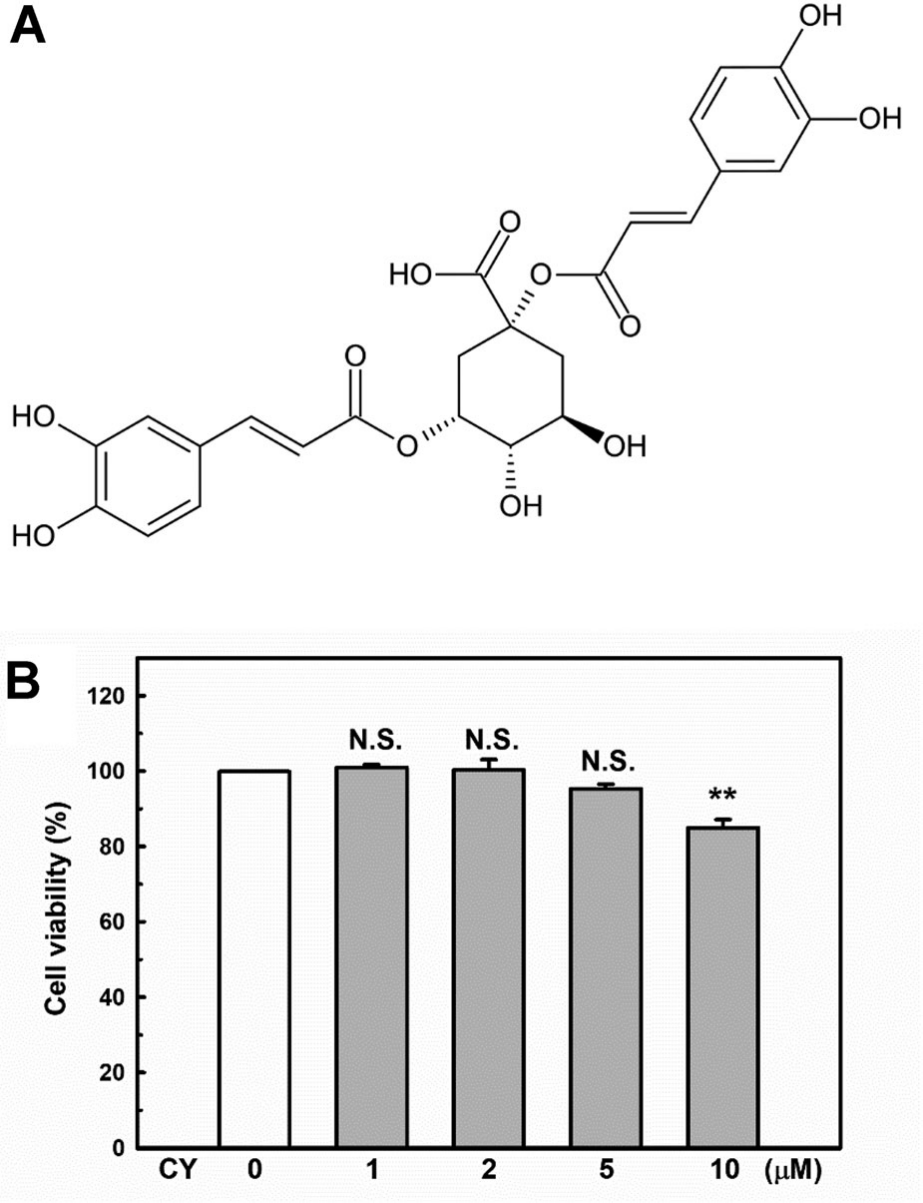
**Chemical structure and cytotoxicity induced by cynarin.** (**A**) Chemical structure of cynarin. (**B**) Cell viability of EA.hy926 cells after treatment with different cynarin concentrations as determined using the cell counting kit-8 assay. The results are presented as the mean ± standard error of the mean (SEM) of three independent experiments. **P < 0.01, compared to the vehicle control group.

The chemical structure of cynarin is shown in Figure 1A. The CCK-8 assay was conducted to explore the cytotoxicity of cynarin on EA.hy926 cells. As shown in Figure 1B, cynarin is safe for use in EA.hy926 cells at doses of 1–5 μM.

**Figure 2.**
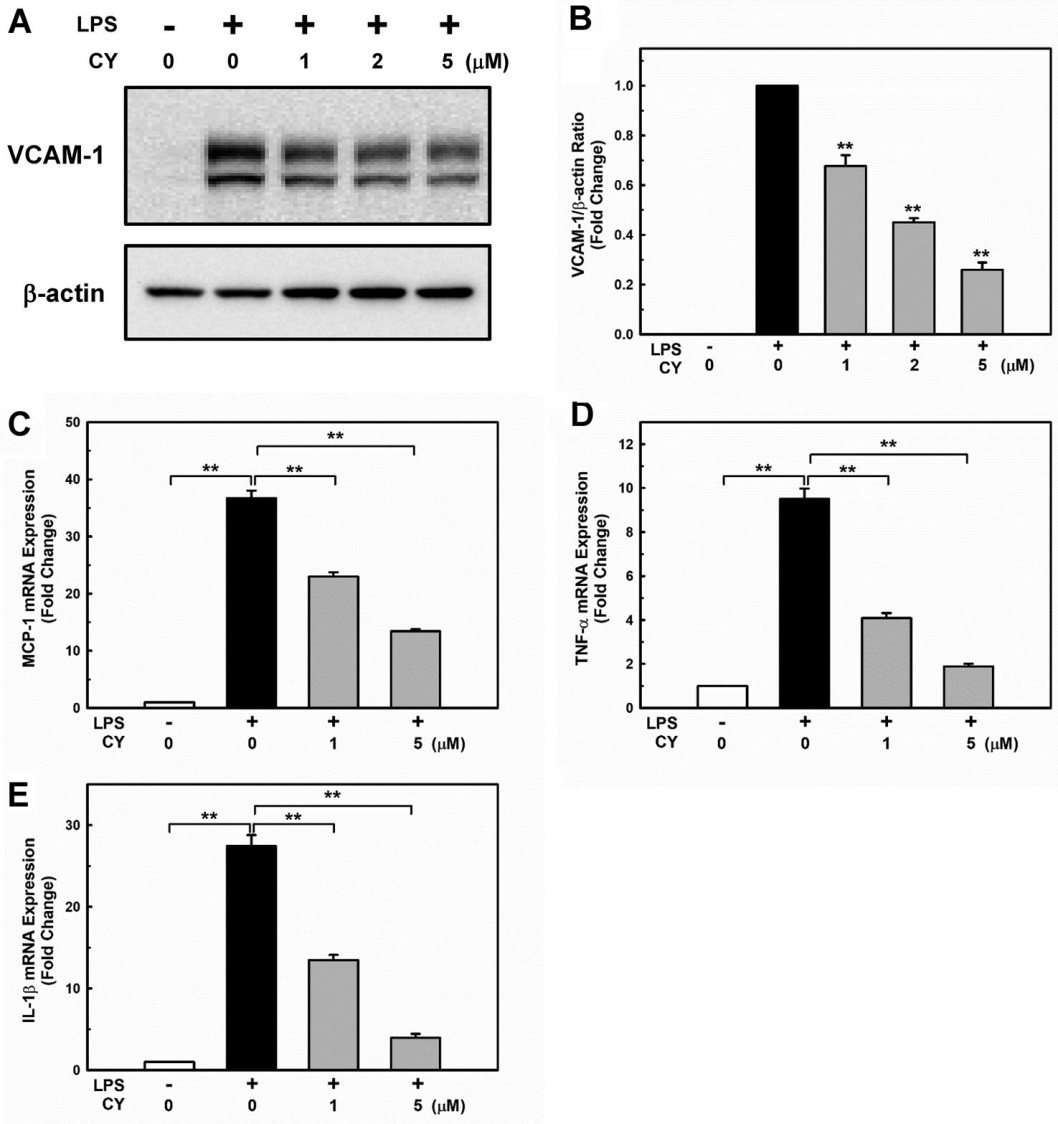
**Effects of cynarin on lipopolysaccharide (LPS)-induced proinflammatory molecule expression in EA.hy926 cells.** EA.hy926 cells were treated with cynarin for 4 h before adding LPS (1 μg/mL) for another 24 h. (**A + B**) VCAM-1 expression level was measured via western blotting. β-actin was considered as a loading control. Data are presented as the mean ± SEM (n = 3). **P < 0.01, compared to the LPS-only group. (**C–E**) The mRNA expression levels of inflammatory genes [monocyte chemoattractant protein-1 (MCP-1), tumor necrosis factor (TNF-α), and interleukin (IL)−1β] as detected via real-time PCR. 18S rRNA was used as an internal control. Data are presented as the mean ± SEM (n = 3). Asterisks indicate significant differences (*P* <   0.01).

Circulating monocytes are the first cells to elicit a proinflammatory response during endotoxemia. These cells respond to extremely low endotoxin (pg/mL) levels (Stoll et al. 2004), while VCAM-1 expression initiates events that lead to monocyte accumulation on the vessel wall (Li et al. 1993). Therefore, the effect of cynarin on VCAM-1 protein and mRNA expression levels in LPS-stimulated EA.hy926 cells was investigated. As shown in Figure 2A + B, LPS exposure significantly increased VCAM-1 expression levels, which were inhibited by cynarin in a dose-dependent manner. To determine whether cynarin could inhibit the expression of proinflammatory cytokines, including monocyte chemoattractant protein-1 (MCP-1), tumor necrosis factor (TNF)-α, and interleukin (IL)−1β, the mRNA levels of proinflammatory cytokines in LPS-treated EA.hy926 cells were examined. Compared with LPS-only treatment, cynarin pretreatment significantly decreased the transcription levels of MCP-1, TNF-α, and IL-1β (Figure 2C–E).

### Cynarin inhibits the LPS-induced activation of p38 MAPK and NF-κB in LPS-stimulated endothelial cells

**Figure 3.**
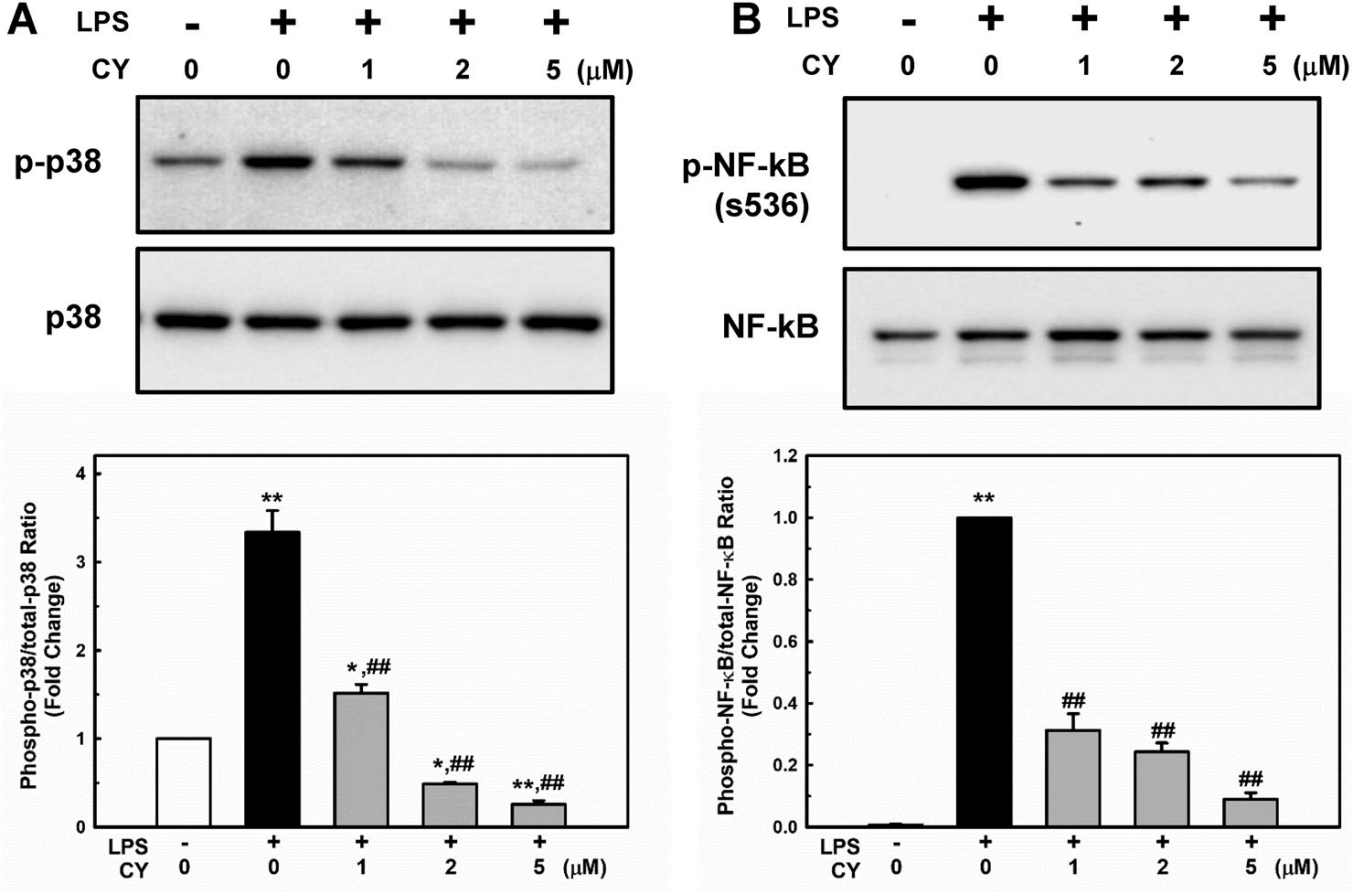
**Effects of cynarin on LPS-stimulated phosphorylation of p38 and NF-κB p65 in EA.hy926 cells.** (**A + B**) The cells were treated with cynarin for 1 h before adding LPS (1 μg/mL) for another 30 min. Relative protein expressions were analyzed via western blotting. The data show the mean ± SEM of three independent experiments. *P < 0.05 and **P < 0.01, compared to the vehicle control group. ^##^*P* < 0.01, compared to the LPS-only group.

MAPKs are essential regulators of endothelial inflammation (Hoefen and Berk 2002). The stimulation of p38 appears to be particularly important (Schumann et al. 1996; Tamura et al. 1998). Additionally, p38 controls the expression of VCAM-1 (Pietersma et al. 1997) and MCP-1 (Goebeler et al. 1999) after cytokine activation. Therefore, we examined whether cynarin inhibited p38 phosphorylation in LPS-treated EA.hy926 cells. As shown in Figure 3A, p38 phosphorylation was significantly increased in LPS-treated cells; however, cynarin pretreatment (1–5 µM) inhibited this effect.

**Figure 4.**
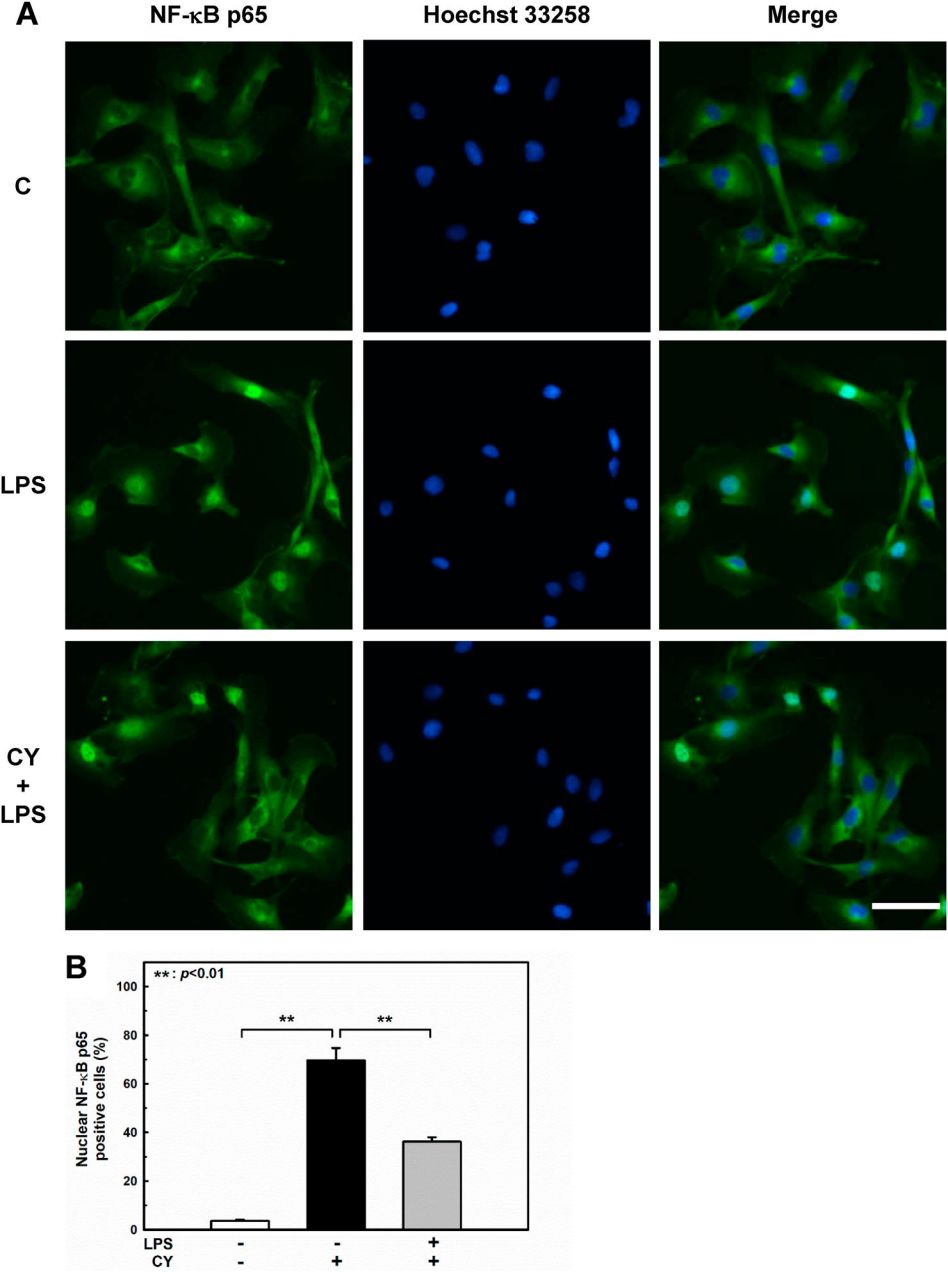
**Effects of cynarin on nuclear translocation of NF-κB p65 in LPS-stimulated EA.hy926 cells.** The cells were treated with cynarin (5 µM) for 1 h before adding LPS (1 μg/mL) for another 30 min. (**A**) Representative immunofluorescence staining images showing NF-κB p65 (green) and the nucleus (blue). Scale bars: 50 µm. (**B**) Quantitation of NF-κB p65 nuclear translocation in the indicated groups. Results are shown as the mean ± SEM (n = 3). Asterisks indicate significant differences (*P* <   0.01).

Activation of NF-κB also regulates gene expression during endothelial activation (Chen et al. 2017). The phosphorylation of NF-κB at Ser536 promotes its nuclear translocation (Sakurai et al. 1999) and facilitates binding to specific promoter sequences (Buss et al. 2012). The influence of cynarin on LPS-induced NF-κB activation in EA.hy926 cells was studied. In LPS-treated cells, phosphorylation of NF-κB p65 was significantly increased; however, pretreatment with cynarin suppressed this effect (Figure 3A). Inhibition of LPS-induced NF-κB p65 activation by cynarin was consistent with its nuclear translocation (Figure 4). These findings suggest that cynarin suppresses the LPS-induced activation of p38 and NF-κB.

### Cynarin inhibits the LPS-induced activation of p38 MAPK and NF-κB in LPS-stimulated endothelial cells

**Figure 5.**
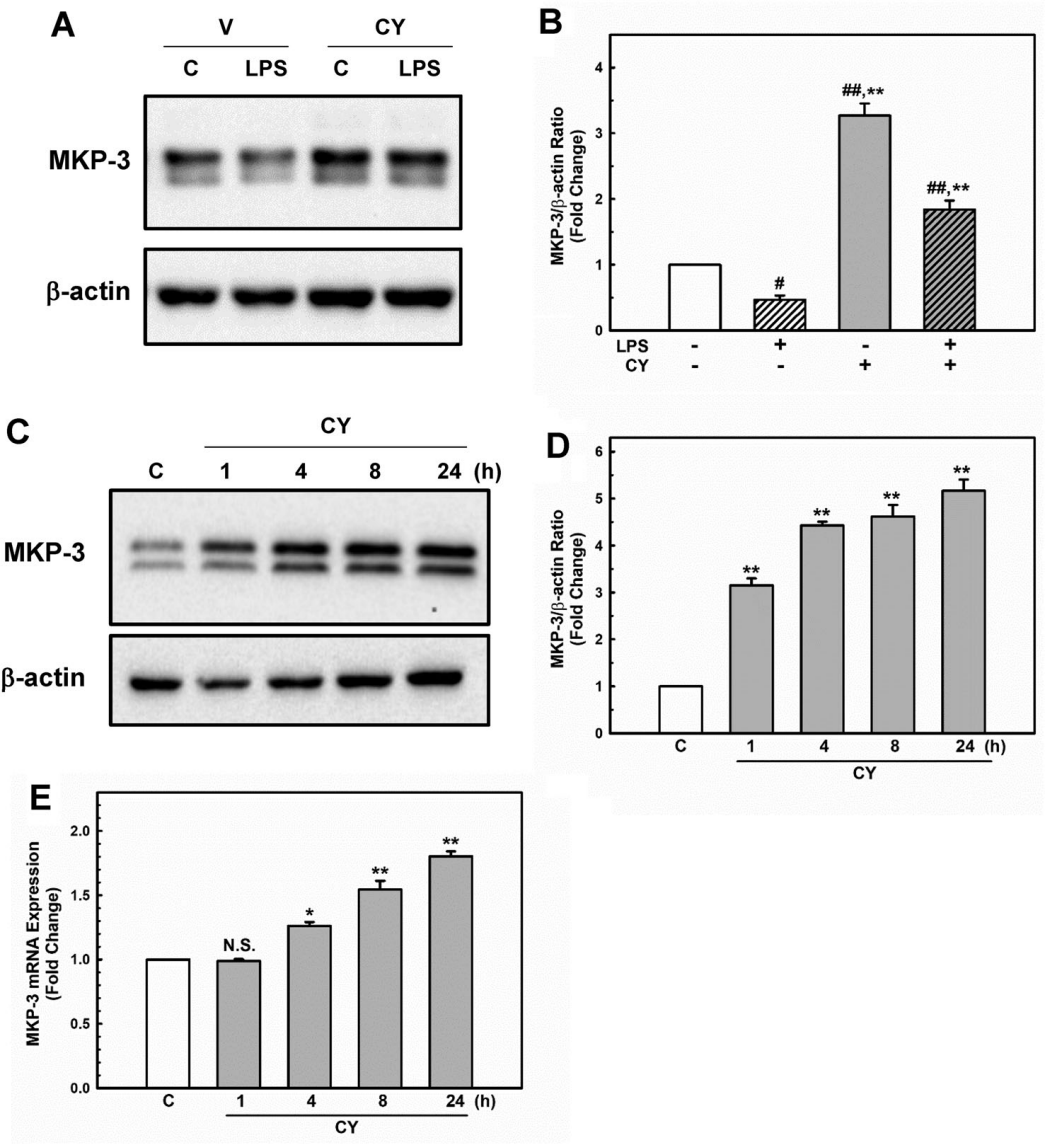
**Effects of cynarin on MKP-3 expression in EA.hy926 cells.** (**A + B**) The cells were treated with cynarin (5 µM) for 1 h before adding LPS (1 μg/mL) for another 30 min. MKP-3 expression was measured via western blotting analysis. β-actin was considered as an internal control. Data are presented as the mean ± SEM (n = 3). **P < 0.01, compared to the LPS-only group. ^#^*P* < 0.05 and ^##^*P* < 0.01, compared to the vehicle control group. The cells were treated with cynarin (5 µM) for 1, 4, 8, or 24 h. Protein expression of MKP-3 was determined using western blotting analysis (**C + D**). MKP-3 mRNA expression was analyzed using real-time PCR (**E**). The data show the mean ± SEM of three independent experiments. *P < 0.05 and **P < 0.01, compared to the vehicle control group.

MKP-3, a member of the MAPK phosphatase family, has been shown to attenuate endothelial inflammatory responses via MAPK dephosphorylation and inactivation (Whetzel et al. 2009; Leng et al. 2018). Recently, we showed that MKP-3 inhibits the p38/NF-κB pathway, which protects against LPS-induced endothelial inflammation (Unenkhuu et al. 2021). Cynarin (Puangpraphant et al. 2011; Liang and Kitts 2018) and MKP-3 (Unenkhuu et al. 2021) have been shown to inhibit p38/NF-κB activation. Therefore, we aimed to determine whether cynarin upregulates MKP-3 expression. Interestingly, EA.hy926 cells pre-incubated with cynarin exhibited a higher level of MKP-3 than the control cells (Figure 5A + B, cynarin only). Moreover, cynarin markedly increased MKP-3 protein (Figure 5C + D) and mRNA (Figure 5E) levels in a time-dependent manner. These findings implied that cynarin positively controls MKP-3 expression, leading to reduced p38 and NF-κB activation.

## Discussion

Low levels of endotoxins induce a mild but persistent induction of proinflammatory mediators, in contrast to high levels of endotoxins, which can elicit a robust and transient expression of proinflammatory mediators (Glaros et al. 2013; Lee et al. 2021). Even after a single meal, a high-fat diet increases LPS leakage from the gut, and individuals with diabetes have been observed to have higher plasma LPS levels (Harte et al. 2012; Gomes et al. 2017). Endothelial cells are the first cells that respond to endotoxemia because they form an interface between the blood flow and the vessel wall (Dauphinee and Karsan 2006). Persistent low-grade endotoxemia has been linked to the development of chronic vascular inflammatory diseases such as atherosclerosis (Wiedermann et al. 1999) and dysfunctional microcirculation (Czabanka et al. 2007). LPS has been hypothesized to play a role in cardiovascular diseases by promoting atherosclerotic plaque formation upon the activation of proinflammatory pathways (Lehr et al. 2001; Vink et al. 2002; Kallio et al. 2008). Additionally, even in low-risk individuals who do not have traditional vascular risk factors, prolonged infections caused by gram-negative bacteria increase the risk of developing atherosclerosis (Wiedermann et al. 1999).

Cynarin is a biologically active functional food component and is the major dicaffeoylquinic acid derivative found in artichokes. It possesses various pharmacological properties, such as free radical scavenging and antioxidant activity (Jimenez-Escrig et al. 2003). In endothelial cells, artichoke extracts reduced oxidative stress induced by inflammatory mediators and oxidized low-density lipoprotein (Zapolska-Downar et al. 2002) and enhanced endothelial nitric oxide synthase (eNOS) expression and NO generation (Li et al. 2004). However, they failed to induce eNOS expression (Li et al. 2004). Xia et al. reported that cynarin exerts an inhibitory effect on inducible NOS (iNOS) expression induced by a cytokine mixture containing interferon (IFN)- γ, IL-1β, and TNF-α in human coronary artery smooth muscle cells (Xia et al. 2014). However, the mechanism by which cynarin inhibits iNOS expression has not been elucidated. Therefore, this study was designed to investigate the anti-inflammatory effects of cynarin in LPS-stimulated endothelial cells.

In the present study, cynarin exhibited anti-inflammatory activity by decreasing VCAM-1 expression level, mediating monocyte recruitment (Gerszten et al. 1998), and lowering proinflammatory cytokine levels. Additionally, cynarin pretreatment markedly abolished p38 activation, which is necessary for VCAM-1 (Pietersma et al. 1997) and MCP-1 (Goebeler et al. 1999) induction in LPS-stimulated endothelial cells. Moreover, cynarin significantly inhibited phosphorylation at Ser536 of NF-κB and the nuclear translocation of NF-κB p65, which is another critical regulator of LPS-induced inflammatory responses. Phosphorylation at Ser536 in the transactivation domain of NF-κB p65 by multiple kinases, including p38 (Rahman et al. 2004; Schmeck et al. 2004), promotes its nuclear translocation (Sakurai et al. 1999) and facilitates inflammatory gene expression (Buss et al. 2012). Artichoke extracts inhibited the activity of NF-κB in acute alcohol-induced liver injury (Tang et al. 2017) and LPS-stimulated THP-1 human leukemic cells (Milackova et al. 2017); therefore, our current results might be in accordance with these previous findings.

MKP-3 inhibits endothelial inflammation (Whetzel et al. 2009; Leng et al. 2018; Unenkhuu et al. 2021); therefore, we examined whether cynarin upregulated MKP-3 expression. As expected, cynarin markedly increased MKP-3 expression level. Interestingly, the decrease in MKP-3 protein levels after LPS treatment was completely recovered by cynarin pretreatment (Figure 5A + B, LPS vs. CY + LPS). This might be due to the antioxidant activity of cynarin, because LPS induces oxidative stress (Zhang et al. 2017) and subsequently induces MKP-3 degradation (Chan et al. 2008). P53 may also mediate MKP-3 induction by cynarin because cynarin-containing artichoke extracts activate p53 (Simsek and Uysal 2013), which has been identified as a transcriptional factor of MKP-3 (Piya et al. 2012). Although endothelial cells also express MKP-1, which regulates the inflammatory response (Furst et al. 2007), it did not affect MKP-1 levels (data not shown), suggesting that it acts through MKP-3 to inhibit LPS-induced inflammation in endothelial cells.

**Figure 6.**
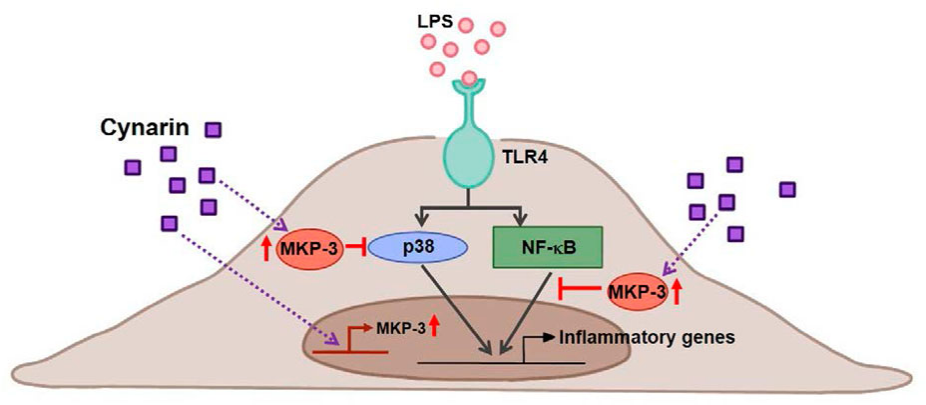
**Diagram of the possible mechanisms of cynarin-induced effects in LPS-stimulated EA.hy926 cells.** Cynarin may function as an MKP-3 inducer to suppress LPS-stimulated inflammation by inhibiting p38 and NF-κB activation.

Overall, the data from the present study suggest that cynarin inhibits endothelial inflammation by inhibiting the p38 and NF-κB pathways by inducing the expression of the negative regulator MKP-3 (Figure 6). Hence, cynarin may be a promising treatment option for various disorders related to endothelial inflammation.
